# Unsteady Boundary Layer Flow and Heat Transfer of a Casson Fluid past an Oscillating Vertical Plate with Newtonian Heating

**DOI:** 10.1371/journal.pone.0108763

**Published:** 2014-10-10

**Authors:** Abid Hussanan, Mohd Zuki Salleh, Razman Mat Tahar, Ilyas Khan

**Affiliations:** 1 Futures and Trends Research Group, Faculty of Industrial Science and Technology, Universiti Malaysia Pahang, Lebuhraya Tun Razak, Kuantan, Pahang, Malaysia; 2 Faculty of Industrial Management, Universiti Malaysia Pahang, Lebuhraya Tun Razak, Kuantan, Pahang, Malaysia; 3 College of Engineering, Majmaah University, Majmaah, Saudi Arabia; China University of Mining and Technology, China

## Abstract

In this paper, the heat transfer effect on the unsteady boundary layer flow of a Casson fluid past an infinite oscillating vertical plate with Newtonian heating is investigated. The governing equations are transformed to a systems of linear partial differential equations using appropriate non-dimensional variables. The resulting equations are solved analytically by using the Laplace transform method and the expressions for velocity and temperature are obtained. They satisfy all imposed initial and boundary conditions and reduce to some well-known solutions for Newtonian fluids. Numerical results for velocity, temperature, skin friction and Nusselt number are shown in various graphs and discussed for embedded flow parameters. It is found that velocity decreases as Casson parameters increases and thermal boundary layer thickness increases with increasing Newtonian heating parameter.

## Introduction

Non-Newtonian fluids are widely used in industries such as chemicals, cosmetics, pharmaceuticals, food and oil & gas [1]. Due to their numerous applications several scientists and engineers are working on them. Despite of the fact non-Newtonian fluids are not as easy as Newtonian fluids. It is due to the fact that in non-Newtonian fluids there does not exist a single constitutive relation that can be used to explain all of them. Therefore several constitutive equations or models are introduced to study their characteristics. The different non-Newtonian models include power law [2], second grade [3], Jeffrey [4], Maxwell [5], viscoplastic [6], Bingham plastic [7], Brinkman type [8], Oldroyd-B [9] and Walters-B [10] models. However, there is another model known as Casson model which is recently the most popular one. Casson [11] was the first who introduce this model for the prediction of the flow behavior of pigment oil suspensions of the printing ink type. Later on, several researchers studied Casson fluid for different flow situations and configurations. Amongst them, Mustafa et al. [12] studied the unsteady flow and heat transfer of a Casson fluid past a moving flat plate. Rao et al. [13] considered the thermal and hydrodynamic slip conditions on heat transfer flow of a Casson fluid past a semi-infinite vertical plate. Heat transfer flow of a Casson fluid past a permeable shrinking sheet with viscous dissipation was considered by Qasim and Noreen [14]. Recently, forced convection flow of a Casson fluid past with surface heat flux over a symmetric porous wedge was investigated by Mukhopadhyay and Mandal [15]. Few other attempts for the Casson fluid can also be found in [16]–[21].

In all these studies mentioned above, the Newtonian heating condition was neglected at the boundary. The situation where the heat is transported to the convective fluid via a bounding surface having finite heat capacity is known as Newtonian heating (or conjugate convective flows). This configuration occurs in convection flows set up when the bounding surfaces absorb heat by solar radiation. Merkin [22] in his pioneering work studied the free convection boundary layer flow past a vertical plate with Newtonian heating. He found the asymptotic solution near the leading edge analytically and the full solution along the whole plate for free convection boundary layer over vertical surfaces numerically. On the other hand, the Newtonian heating situation occurs in many important engineering devices, such as heat exchanger and conjugate heat transfer around fins. Therefore, in view of such applications several authors have used the Newtonian heating condition in their convective heat transfer problems and have obtained the solutions either numerically [23]–[26] or analytical forms [27]–[33].

Most of the existing studies on unsteady boundary layer flow and heat transfer with Newtonian heating condition are limited to the Newtonian fluid or they are solved using any numerical or approximate technique. This motivates us to consider the Newtonian heating phenomenon in the present work for non-Newtonian fluids. More exactly, our aim is to investigate unsteady boundary layer flow and heat transfer of a Casson fluid past an infinite oscillating vertical plate with Newtonian heating condition. The equations of the problem are first formulated and then transformed into their dimensionless forms where the Laplace transform method is applied to find the exact solutions for velocity and temperature.

## Mathematical Formulation

**Figure 1:**
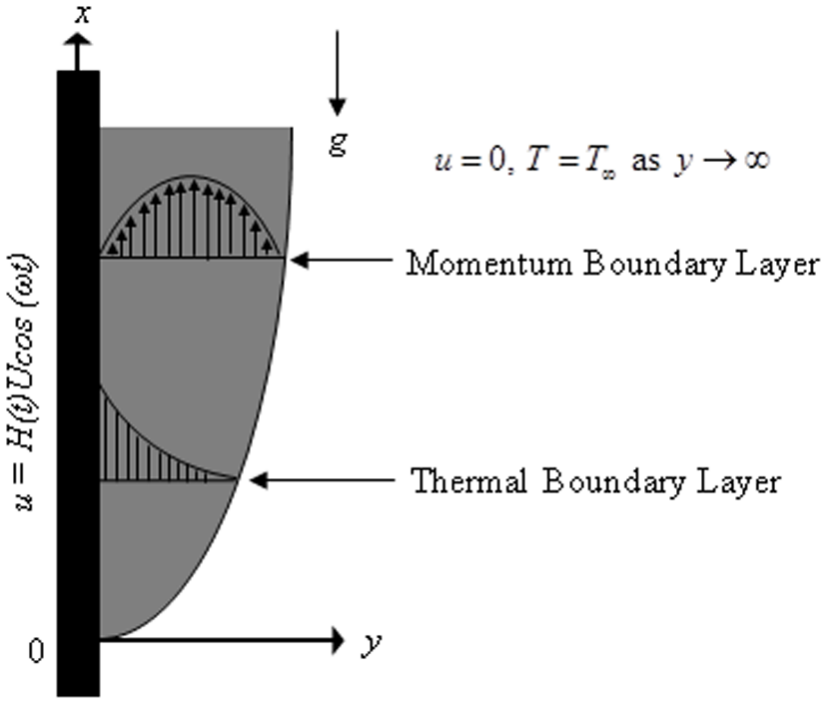
Physical model and coordinate system.

Let us consider the heat transfer effect on unsteady boundary layer flow in a Casson fluid past an infinite oscillating vertical plate fixed at 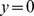, the flow being confined to 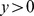, where 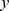 is the coordinate axis normal to the plate. Initially, for time 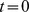, both plate and fluid are at stationary condition with the constant temperature 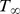. At time 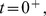 the plate started an oscillatory motion in its plane 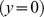 according to
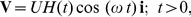
(1)where 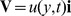, 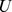 is the amplitude of the motion, 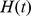
is the unit step function, 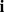 is the unit vector in the vertical flow direction and 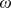 is the frequency of plate oscillation. At the same time, the heat transfer from the plate to the fluid is proportional to the local surface temperature 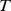. We assume that the rheological equation for an isotropic and incompressible Casson fluid, reported by Casson [11], is
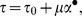
equivalently



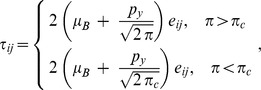
where 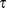 is the shear stress, 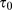 is the Casson yield stress, 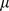 is the dynamic viscosity, 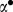 is the shear rate, 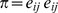 and 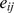 is the 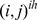 component of the deformation rate, 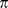 is the product of the component of deformation rate with itself, 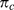 is a critical value of this product based on the non-Newtonian model, 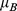 the is plastic dynamic viscosity of the non-Newtonian fluid and 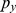 the is yield stress of fluid. Under these assumptions the unsteady boundary layer flow with heat transfer is governed by momentum and energy equations: 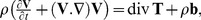
(2)

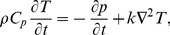
(3)where 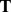 is the Cauchy stress tensor, 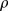 is the fluid density, 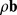 is the body force, *p* is the pressure, 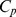 is the heat capacity at constant pressure and 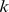 is the thermal conductivity. Under the Boussinesq approximation along with the assumption that the pressure is uniform across the boundary layer, we get the following set of partial differential equations:
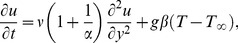
(4)

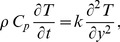
(5)with following initial and boundary conditions 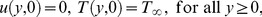
(6)

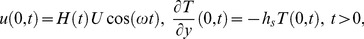
(7)

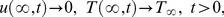
(8)in which 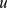 is the axial velocity, 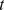 is the time, 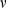
is the kinematic viscosity, 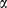 is the Casson fluid parameter, 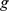 is the acceleration due to gravity, 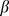 is the volumetric coefficient of thermal expansion and 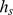 is the heat transfer coefficient. The geometry of the problem is presented in Figure 1.

To reduce the above equations into their non-dimensional forms, we introduce the following non-dimensional quantities
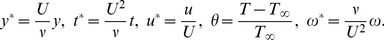
(9)Substituting equation (9) into equations (4) and (5), we obtain the following non-dimensional partial differential equations (^*^ symbols are dropped for simplicity)
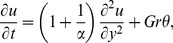
(10)

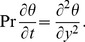
(11)The corresponding initial and boundary conditions in non-dimensional form are
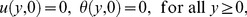
(12)

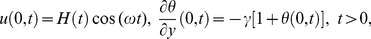
(13)

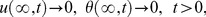
(14)where
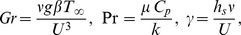
are the Grashof number, the Prandtl number and the conjugate parameter for Newtonian heating respectively. We note that equation (13) gives 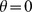 when 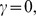 corresponding to having 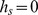 and hence no heating from the plate exists [23], [32].

## Method of Solution

In order to obtain the exact solution of the present problem, we will use the Laplace transform technique. Applying the Laplace transforms with respect to time 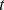 to the equations (10)–(11), we get
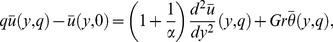
(15)

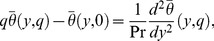
(16)Here, 
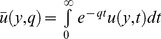
 and 
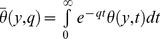
 denote the Laplace transforms of 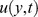 and 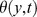, respectively. Using the initial condition (12), we get
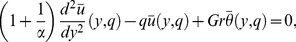
(17)

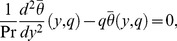
(18)The corresponding transformed boundary conditions are
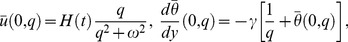
(19)

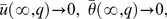
(20)The solutions of equations (17) and (18) subject to the boundary conditions (19) and (20) are
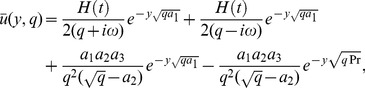
(21)

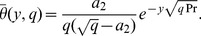
(22)By taking the inverse Laplace transforms of above equations, we obtain 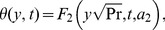
(23)

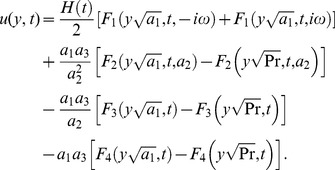
(24)The solution for velocity given in equation (24) is not valid, when 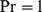 and 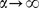. In this case, the solution obtained is given by
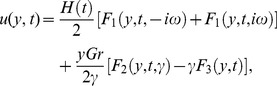
(25)where
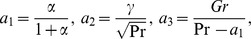


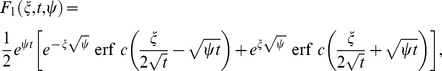


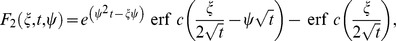


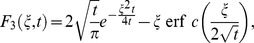


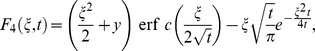


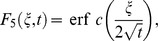


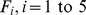
, are dummy functions of the dummy variables 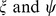.

The dimensionless expression for skin friction evaluated from equation (24) is given by
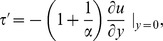


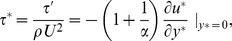


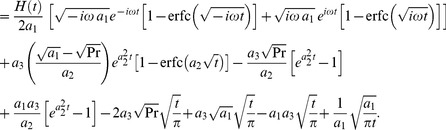
(26)where 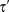 is the dimensional skin friction. The dimensionless expression of Nusselt number is given by
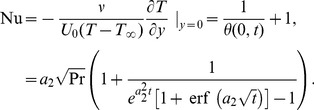
(27)


## Limiting Cases

The solutions obtained here are more general. In this section, we consider some of their limiting cases.

### Solution in case of Newtonian fluid

If 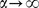, the solution for velocity given in equation (24) reduces to the corresponding solution for Newtonian fluid given by
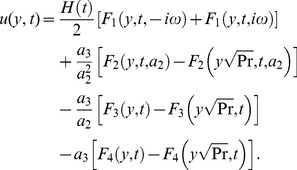
(28)It is important to note that the above solution (28) for Newtonian fluid over an impulsively moved plate when 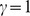 is similar to that obtained by [27].

### Solution in the absence of free convection

In the absence of free convection, which is numerically corresponds to 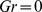, the equation (24) reduces to 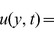
(29)


### Solution of Stokes first problem

By making 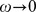 into equation (24), we get the classical solution
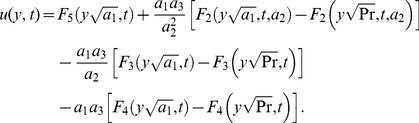
(30)corresponding to the Stokes first problem for Casson fluid over an impulsively motion of the plate.

## Graphical Results and Discussion

Exact solutions for the problem of unsteady boundary layer heat transfer flow of an incompressible Casson fluid past an infinite oscillating vertical plate with Newtonian heating condition are obtained. For the physical behavior of embedded parameters such as Casson parameter 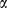, Prandtl number 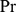, Grashof number 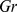, conjugate parameter for Newtonian heating 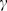, time 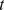 and phase angle 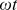, these solutions are plotted in graphs (Figures 2–15) and discussed in details.

**Figure 2:**
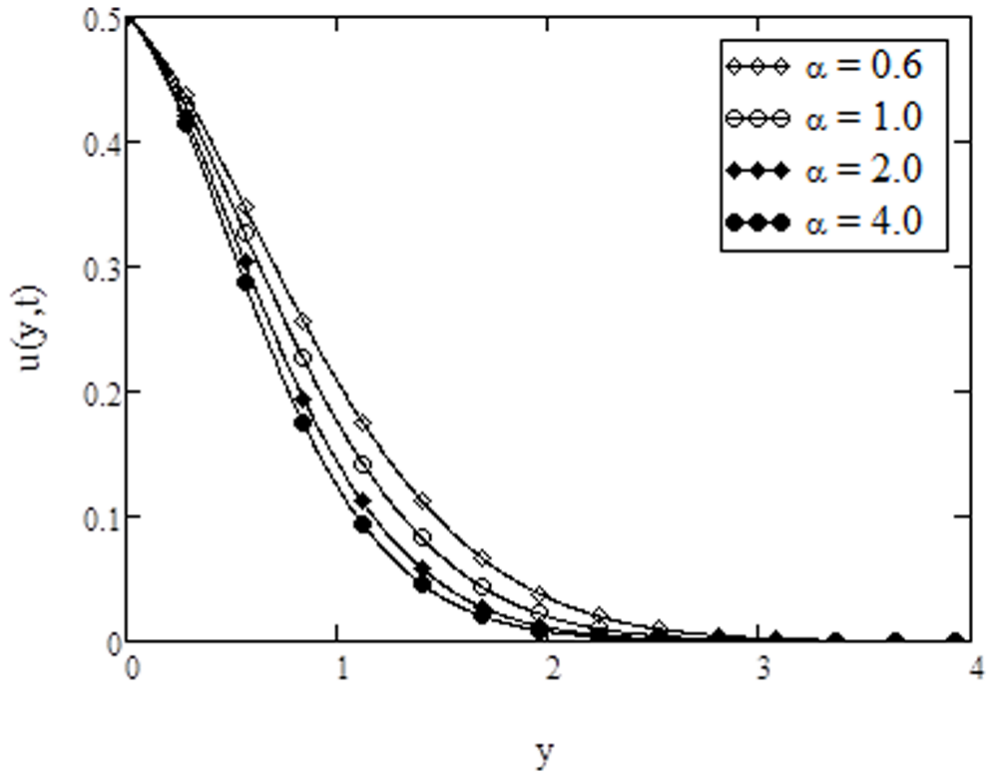
Velocity profiles for different values of 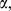
 **when** 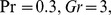 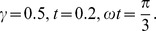

**Figure 3:**
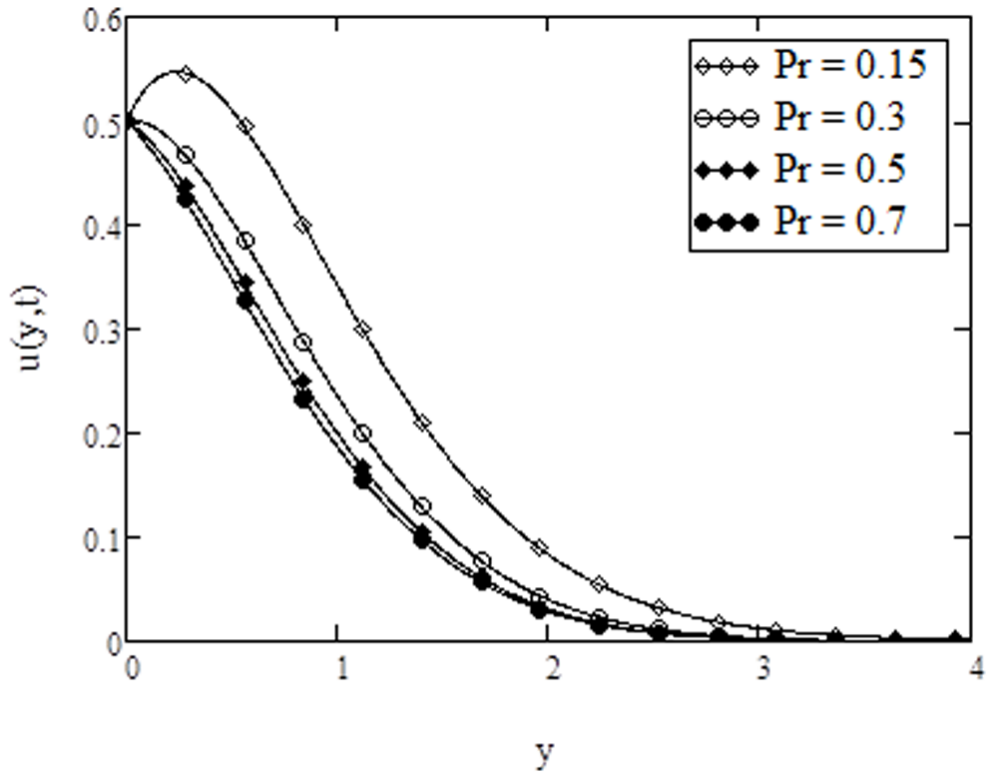
Velocity profiles for different values of 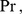
 **when** 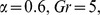 **** 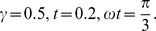

**Figure 4:**
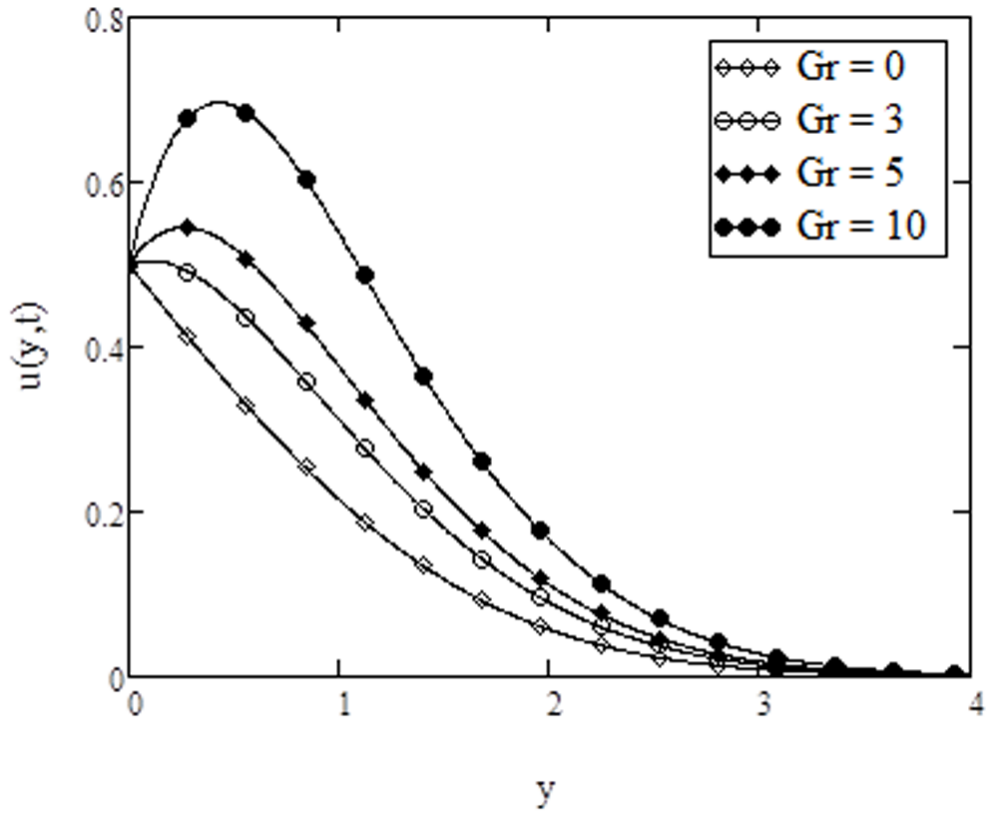
Velocity profiles for different values of 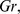
 **when** 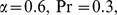 **** 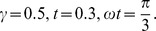

**Figure 5:**
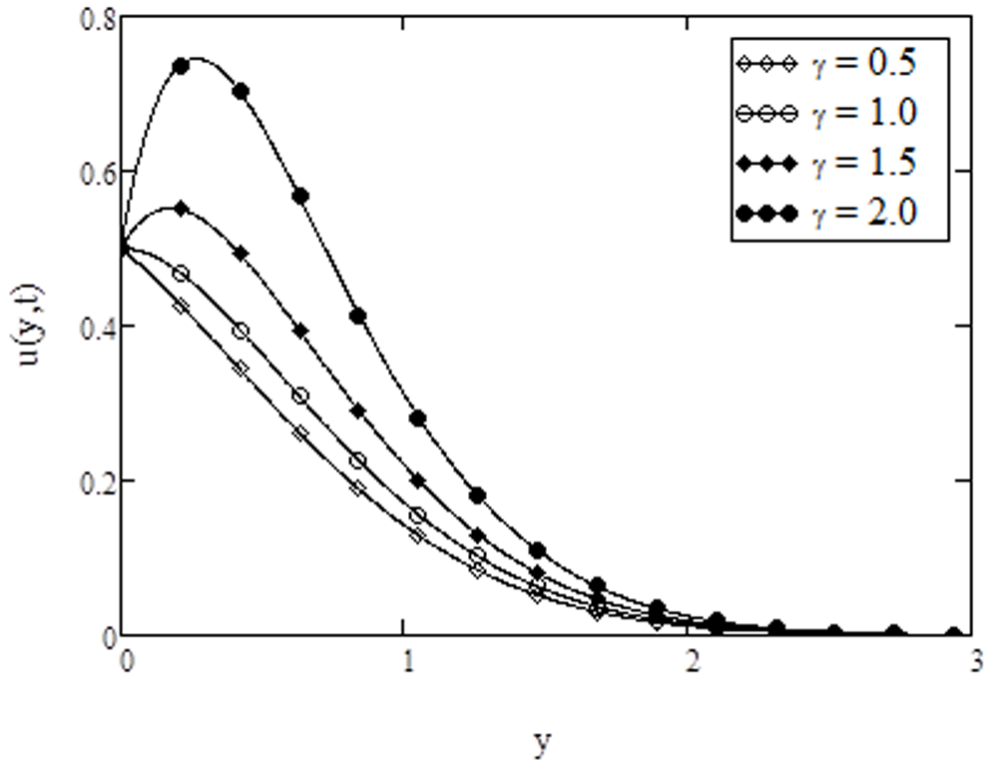
Velocity profiles for different values of 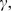
 **when** 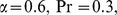 **** 
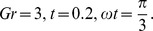

**Figure 6:**
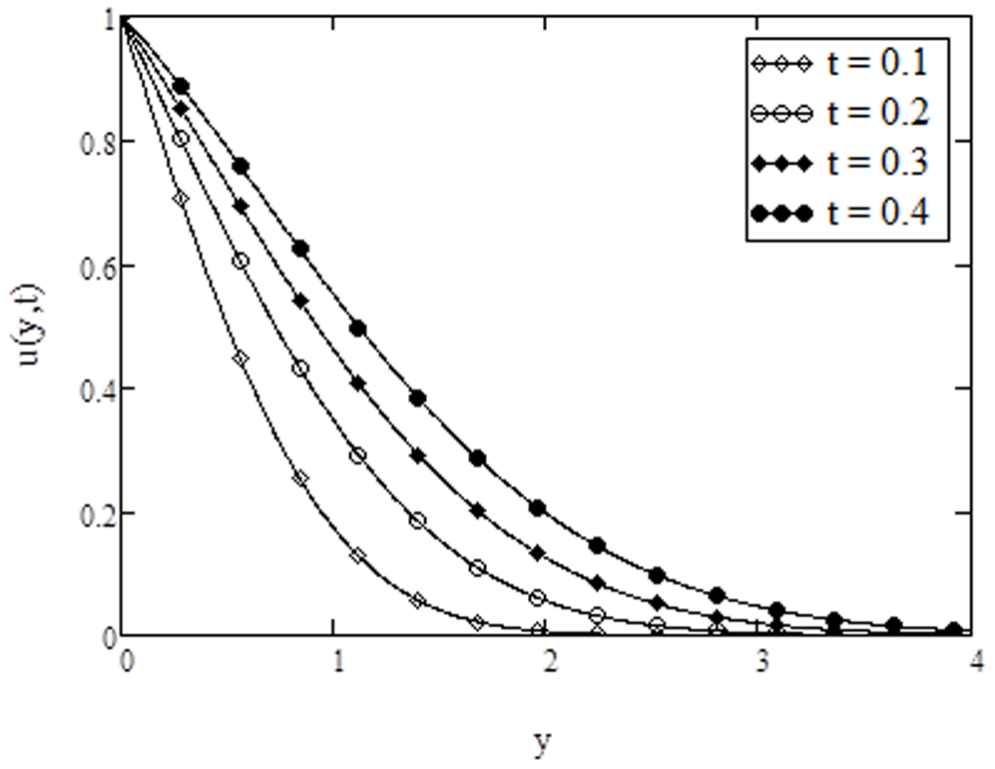
Velocity profiles for different values of 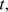
 **when** 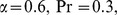 **** 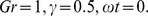

**Figure 7:**
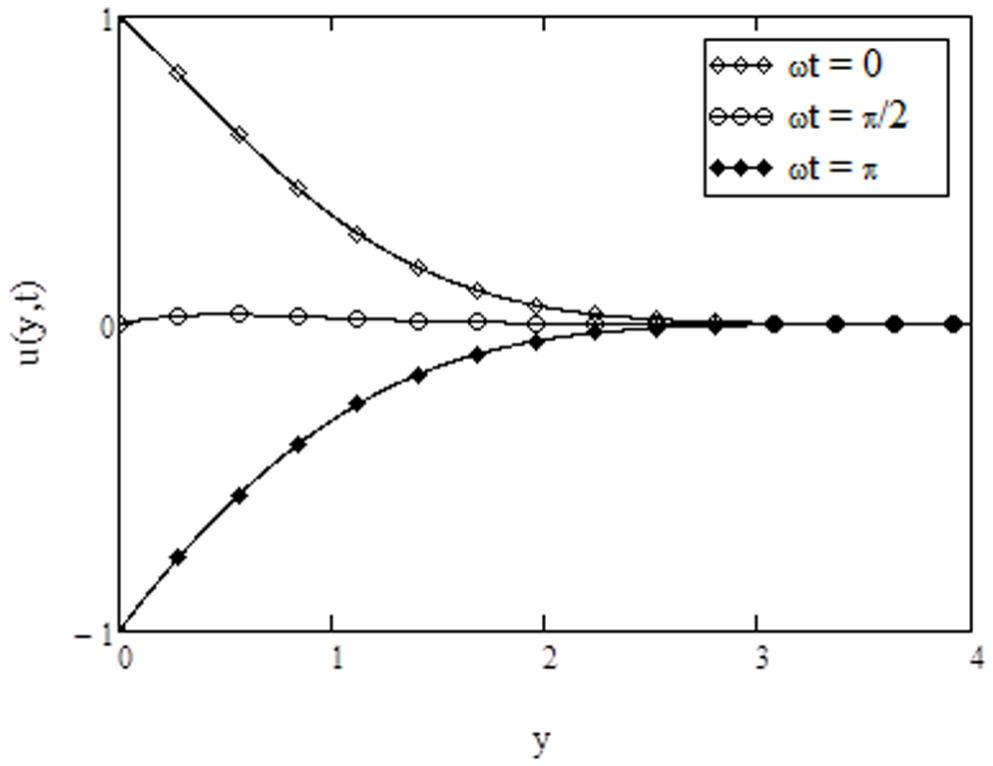
Velocity profiles for different values of 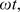
 **when** 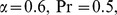 **** 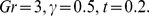

**Figure 8:**
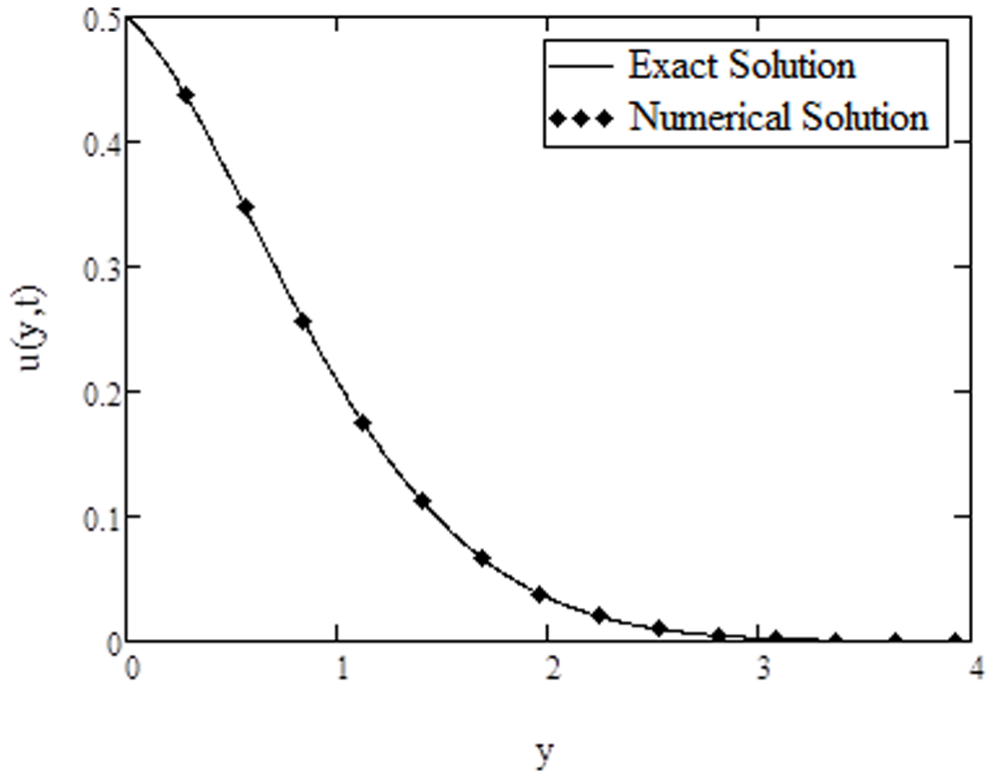
Comparison of exact solution of velocity with numerical solution, when 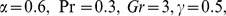
 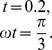

**Figure 9:**
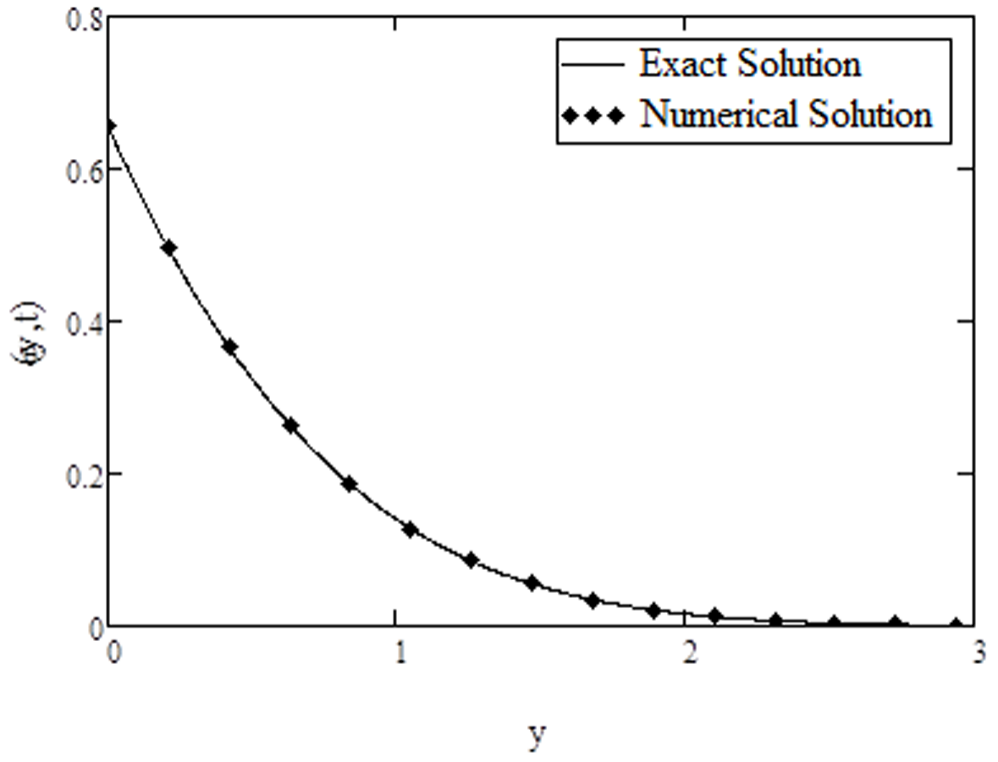
Comparison of exact solution of temperature with numerical solution, when 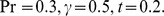

**Figure 10:**
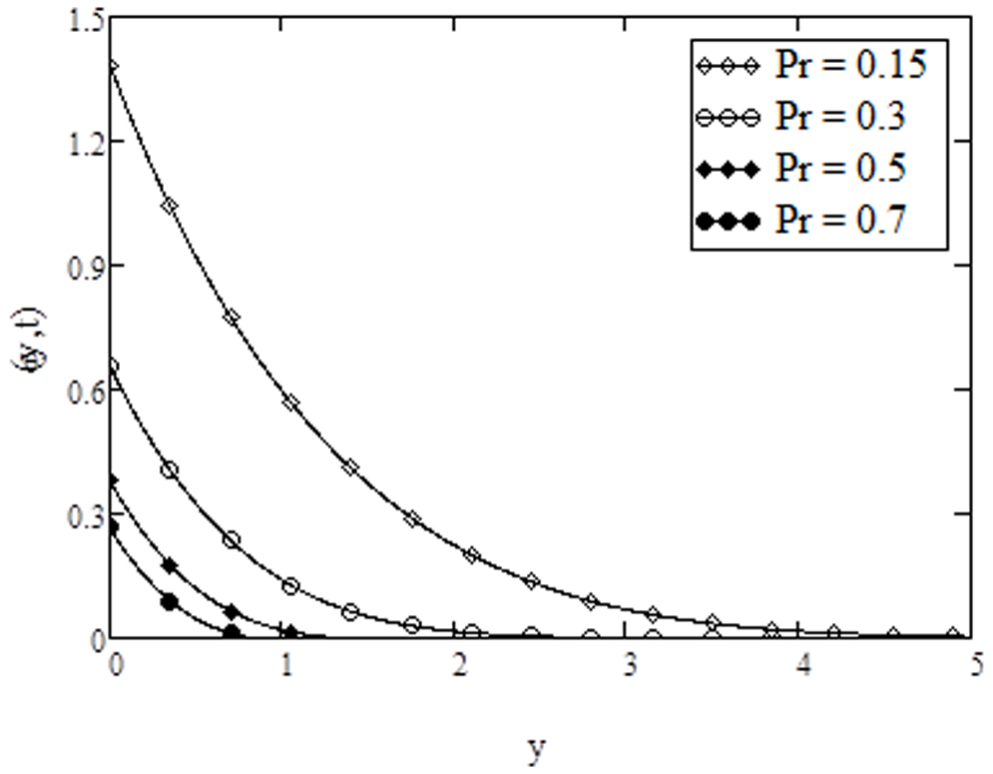
Temperature profiles for different values of 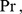
 **when** 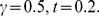

**Figure 11:**
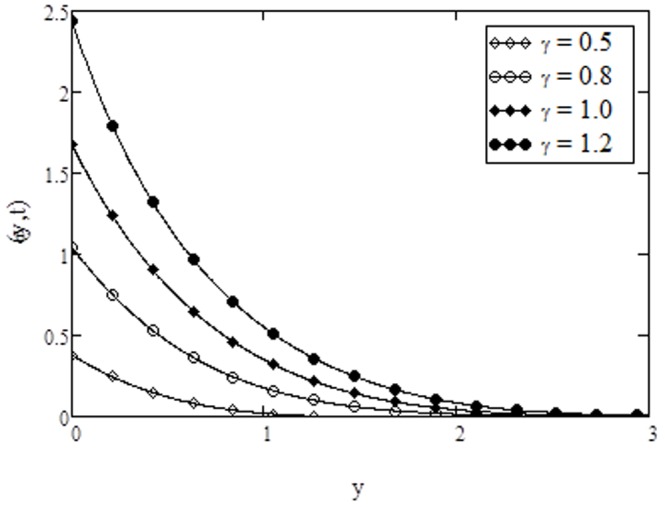
Temperature profiles for different values of 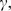
 **when** 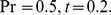

**Figure 12:**
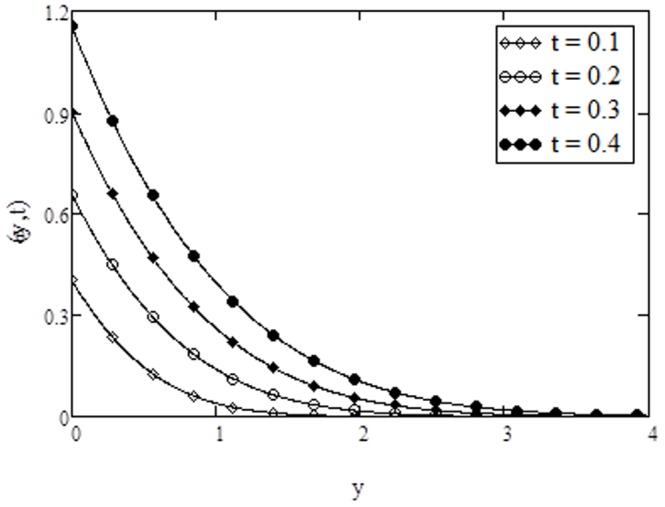
Temperature profiles for different values of 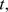
 **when** 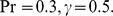

**Figure 13:**
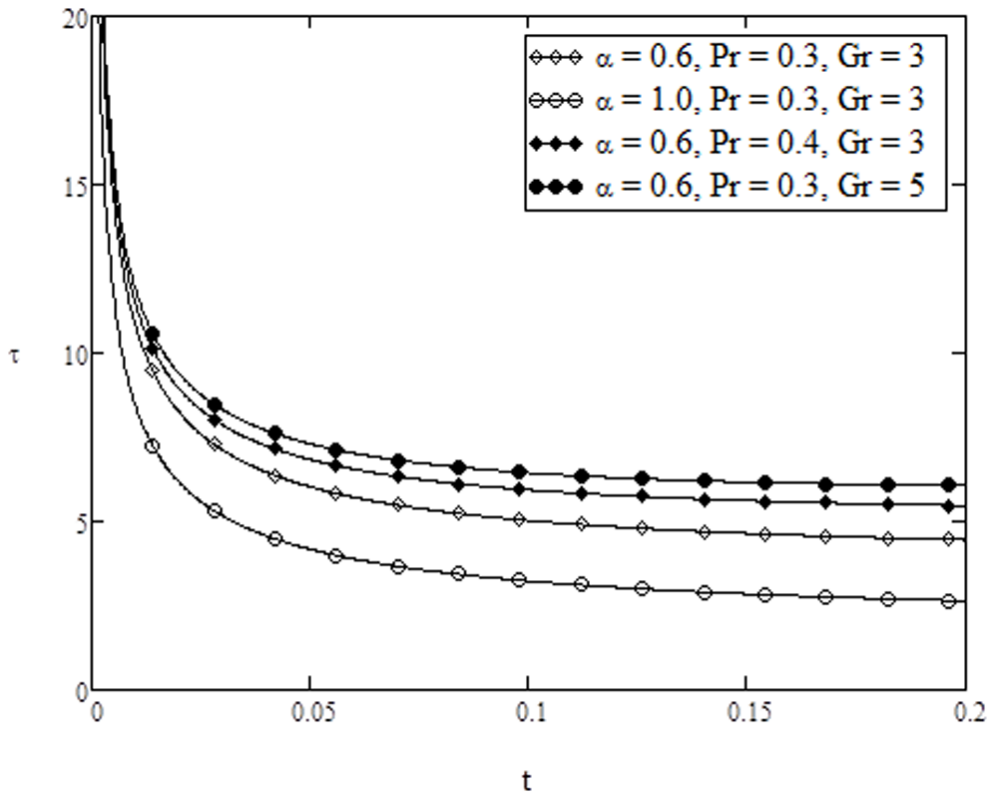
Skin-friction variation for different values of 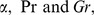
 **when** 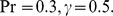

**Figure 14:**
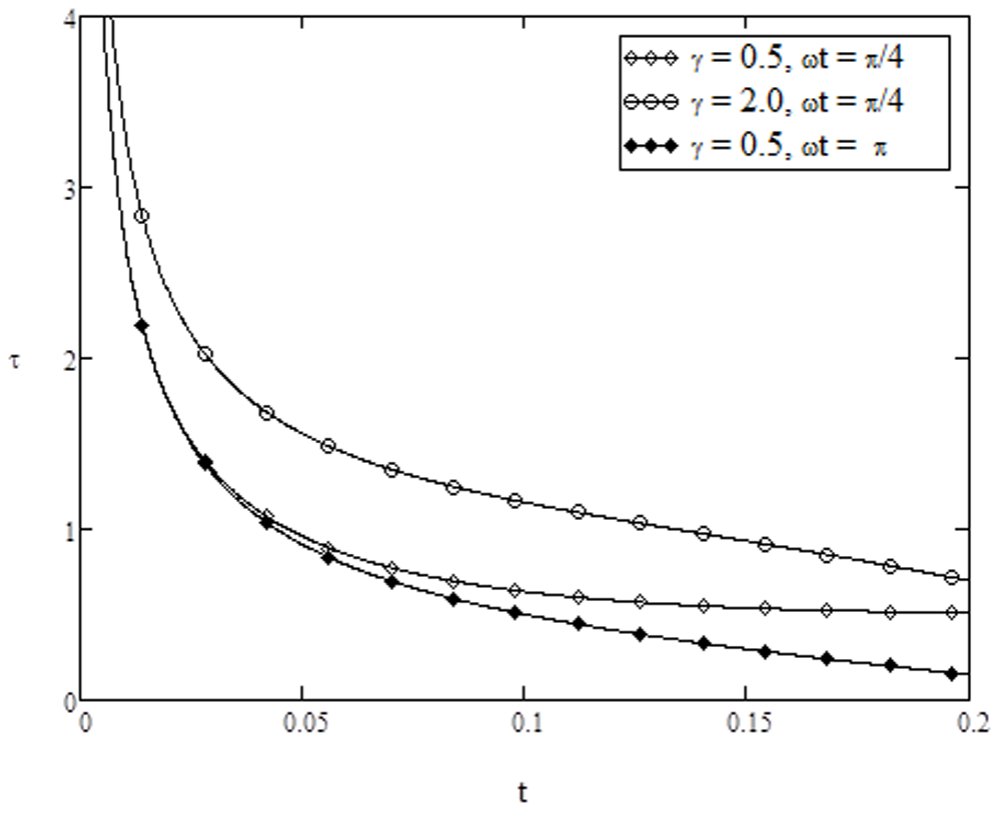
Skin-friction variation for different values of 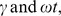
 **when** 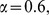 **** 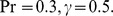

**Figure 15:**
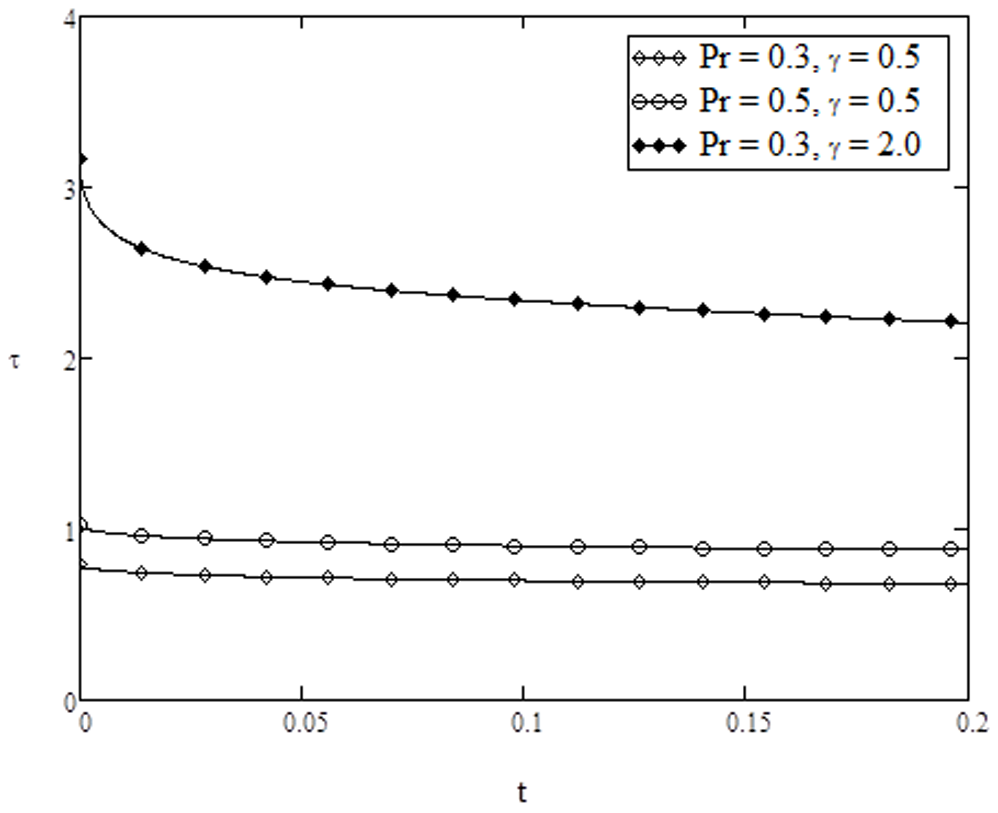
Nusselt number variation for different values of 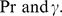

The velocity profiles for different values of Casson parameter 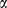 are shown in Figure 2. From this figure, it is observed that velocity decreases with increasing values of 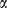. Further, it is noticed that Casson parameter does not have any influence as the fluid moves away from the bounding surface. The velocity profiles are shown in Figure 3 for different values of Prandtl number 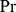. It is observed that velocity decreases with increasing Prandtl number. This situation is in consistence with the physical observation because fluids with large Prandtl number have high viscosity and small thermal conductivity, which makes the fluid thick and hence causes a decrease in velocity of fluid. In addition, the curves show that velocity of fluid is maximum near the plate and approaches to zero as 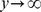 (for away from the plate). It is also found from Figures 2 and 3, that the behavior of 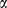 and 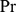 on the velocity profiles are quite identical with that found in figure 7 and 9, of Rao et al. [13]. The effects of Grashof number 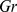 on the velocity profiles are shown in Figure 4. The trend shows that velocity increases with increasing values of 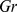. It is true physically also because the role of Grashof number in heat transfer flow is to increase the strength of the flow. Here 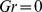 corresponds to the absence of free convection, while 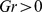 represents to the cooling problem. Moreover, the cooling problem is of great importance and mostly encountered in engineering applications, such as in the cooling of electronic components and nuclear reactors. For different values of conjugate parameter for Newtonian heating 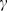, the velocity profiles are plotted in Figure 5. An increase in conjugate parameter for Newtonian heating may reduce the fluid density and increases the momentum boundary layer thickness, as a result, the velocity increases within the boundary layer. Further, the behavior of Grashof number and conjugate parameter on the velocity profiles are quite identical with that found in figures 7 and 8 of Jain [33]. Figure 6 demonstrates the effect of time 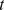 on the velocity profiles. It is found that velocity increases with an increase in 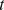. The velocity profiles for different values of phase angle 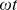 are depicted in Figure 7. It is found that the velocity shows an oscillatory behavior. The oscillations near the plate are of great significance; however, these oscillations reduce for large values of the independent variable 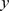 and approach to zero as 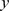 approaches to infinity. The numerical results for velocity and temperature are computed in Table 1 and Table 2 respectively. Furthermore, Figures 8 and 9 are prepared to show the comparison of the present analytical results for velocity and temperature given by equations (24) and (23) with the numerical results in Table 1 and Table 2. It is found that the analytical results are quite identical with the numerical results.

**Table 1:** Numerical results for velocity.

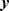	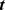	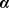	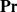	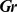	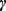	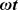	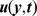
0	0.2	0.6	0.3	3	0.5	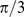	0.500
1	0.2	0.6	0.3	3	0.5	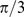	0.208
2	0.2	0.6	0.3	3	0.5	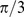	0.035
1	0.4	0.6	0.3	3	0.5	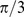	0.417
2	0.4	0.6	0.3	3	0.5	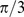	0.162
1	0.2	1.0	0.3	3	0.5	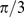	0.176
2	0.2	1.0	0.3	3	0.5	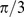	0.020
1	0.2	0.6	0.5	3	0.5	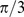	0.187
2	0.2	0.6	0.5	3	0.5	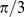	0.029
1	0.2	0.6	0.3	5	0.5	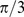	0.236
2	0.2	0.6	0.3	5	0.5	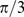	0.040
1	0.2	0.6	0.3	3	1.0	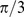	0.283
2	0.2	0.6	0.3	3	1.0	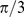	0.047
1	0.2	0.6	0.3	3	0.5	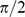	0.042
2	0.2	0.6	0.3	3	0.5	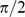	0.008

**Table 2:** Numerical results for temperature.

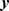	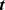	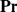	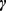	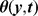
0	0.2	0.3	0.5	0.656
1	0.2	0.3	0.5	0.140
2	0.2	0.3	0.5	0.016
0	0.4	0.3	0.5	1.155
1	0.4	0.3	0.5	0.396
0	0.2	0.5	0.5	0.379
1	0.2	0.5	0.5	0.020
0	0.2	0.3	1.0	2.827
1	0.2	0.3	1.0	0.893

The variation of temperature for different values of Prandtl number 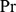 are plotted in Figure 10. It is found that temperature of the fluid decreases with increasing values of 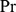. This is in agreement with the physical fact that with increasing Prandtl number, the viscosity of the fluid increases, the fluid become more thick which reduces the heat transfer. From Figure 11, it is observed that an increase in the conjugate parameter for Newtonian heating increases the thermal boundary layer thickness and as a result the surface temperature of the plate increases. It is also observed that there is a sharp rise in temperature with the increase of conjugate parameter. Note that the variations in temperature due to conjugate parameter are identical to the published work of [31], [33]. It is observed from Figure 12 that the fluid temperature increases with an increase in time 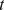.

On the other hand, variation of skin friction and Nusselt number verses time are plotted in Figures 13–15 for various parameters of interest. It is found from Figure 13 that skin friction increases with increasing value of 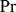 whereas it decreases with increasing value of 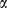 and 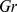, when 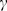 and 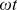 are fixed. From Figure 14, it is noticed that the skin friction increases with increasing values of conjugate parameter 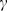, while reverse effect is observed for phase angle 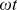. Finally, the Nusselt number increases as 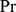 and 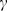 are increased as shown in Figure 15. Finally, for the comparison of the present results with those existing in the literature we have plotted Table 3. It is found that for 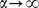, our results are quite identical with those obtained in [32], when 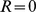
(in the absence of thermal radiation).

**Table 3:** Comparison of skin friction calculated in the present work at 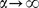 and in [32], when 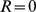.

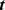	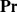	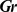	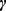	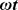	Present Results	Results of [32]
0.01	0.35	5	1	0	5.5818	5.5818
0.02	0.35	5	1	0	3.8620	3.8620
0.01	0.50	5	1	0	5.5956	5.5956
0.01	0.35	10	1	0	5.5218	5.5218
0.01	0.35	5	2	0	5.5027	5.5027
0.01	0.35	5	1	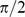	4.4819	4.4819

## Conclusions

In this paper, exact solutions of unsteady boundary layer flow and heat transfer of a Casson fluid past an oscillating vertical plate with Newtonian heating are obtained using the Laplace transform technique. The results obtained have shown that the effect of number increases the velocity but reduces the skin friction. However, the velocity is decreased when the Casson parameter is increased. Moreover, in the particular case of Newtonian fluid, the analytical results obtained in the present work were compared with those available in the literature, obtaining an excellent agreement with those given in [32]. A significant finding of this study is that flow separation can be controlled by increasing the value of Casson fluid parameter as well as by increasing Prandtl number.

## References

[1] Chaoyang W, Chuanjing T (1989) Boundary layer flow and heat transfer of non-Newtonian fluids in porous media. International Journal of Heat and Fluid Flow (10): 160–165.

[2] Olajuwon BI (2009) Flow and natural convection heat transfer in a power law fluid past a vertical plate with heat generation. International Journal of Nonlinear Science (7): 50–56.

[3] Hayat T, Khan I, Ellahi R, Fetecau C (2008) Some unsteady MHD flows of a second grade fluid through porous medium. Journal Porous Media (11): 389–400.

[4] Qasim M (2013) Heat and mass transfer in a Jeffrey fluid over a stretching sheet with heat source/sink. Alexandria Engineering Journal (52): 571–575.

[5] Khan I, Farhad A, Samiulhaq, Sharidan S (2013) Exact solutions for unsteady MHD oscillatory flow of a Maxwell fluid in a porous medium. Zeitschrift Fur Naturforschung A (68): 635–645.

[6] Hassan MA, Pathak M, Khan MK (2013) Natural convection of viscoplastic fluids in a square enclosure. Journal of Heat Transfer (135): 122501–12.

[7] Kleppe J, Marner WJ (1972) Transient free convection in a Bingham plastic on a vertical flat plate. Journal of Heat Transfer (1972): 371–376.

[8] Zakaria MN, Abid H, Khan I, Sharidan S (2013) The effects of radiation on free convection flow with ramped wall temperature in Brinkman type fluid. Jurnal Teknologi (62): 33–39.

[9] Khan I, Fakhar K, Anwar MI (2012) Hydromagnetic rotating flows of an Oldroyd-B fluid in a porous medium. Special Topics and Review in Porous Media (3): 89–95.

[10] Khan I, Farhad A, Sharidan S, Qasim M (2014) Unsteady free convection flow in a Walters-B fluid and heat transfer analysis. Bulletin of the Malaysian Mathematical Sciences Society (37): 437–448.

[11] Casson N (1959) A flow equation for pigment oil suspensions of the printing ink type. In: Rheology of disperse systems. Mill CC (Ed.) Pergamon Press, Oxford 84–102.

[12] Mustafa M, Hayat T, Pop I, Aziz A (2011) Unsteady boundary layer flow of a Casson fluid due to an impulsively started moving flat plate. Heat Transfer-Asian Research (40): 553–576.

[13] Rao AS, Prasad VR, Reddy NB, Beg OA (2013) Heat transfer in a Casson rheological fluid from a semi-infinite vertical plate with partial slip. Heat Transfer-Asian Research (2013): 1–20.

[14] Qasim M, Noreen S (2014) Heat transfer in the boundary layer flow of a Casson fluid over a permeable shrinking sheet with viscous dissipation. The European Physical Journal Plus (129): 1–8.

[15] Mukhopadhyay S, Mandal IS (2014) Boundary layer flow and heat transfer of a Casson fluid past a symmetric porous wedge with surface heat flux. Chinese Physics B (23): 044702–6.

[16] Venkatesan J, Sankar DS, Hemalatha K, Yatim Y (2013) Mathematical analysis of Casson fluid model for blood rheology in stenosed narrow arteries. Journal of Applied Mathematics (2013): 1–11.

[17] Malik MY, Naseer M, Nadeem S, Rehman A (2013) The boundary layer flow of Casson nanofluid over a vertical exponentially stretching cylinder. Applied Nanoscience doi 10.1007/s13204-013-0267-0

[18] Mukhopadhyay S, Bhattacharyya K, Hayat T (2013) Exact solutions for the flow of Casson fluid over a stretching surface with transpiration and heat transfer effects. Chinese Physics B (22): 114701–6.

[19] Pramanik S (2014) Casson fluid flow and heat transfer past an exponentially porous stretching surface in presence of thermal radiation. Ain Shams Engineering Journal (5): 205–212.

[20] Kirubhashankar CK, Ganesh S (2014) Unsteady MHD flow of a Casson fluid in a parallel plate channel with heat and mass transfer of chemical reaction. Indian Journal of Research (3): 101–105.

[21] Shehzad SA, Hayat T, Qasim M, Asghar S (2013) Effects of mass transfer on MHD flow of Casson fluid with chemical reaction and suction. Brazilian Journal of Chemical Engineering (30): 187–195.

[22] Merkin JH (1994) Natural convection boundary layer flow on a vertical surface with Newtonian heating. International Journal of Heat and Fluid Flow (15): 392–398.

[23] Salleh MZ, Nazar R, Pop I (2010) Boundary layer flow and heat transfer over a stretching sheet with Newtonian heating. Journal of the Taiwan Institute of Chemical Engineers (41): 651–655.

[24] Salleh MZ, Nazar R, Arifin NM, Pop I (2011) Forced convection heat transfer over a circular cylinder with Newtonian heating. Journal of Engineering Mathematics (69): 101–110.

[25] Das S, Mandal C, Jana RN (2012) Radiation effects on unsteady free convection flow past a vertical plate with Newtonian heating. International Journal of Computer Applications (41): 36–41.

[26] Kasim ARM, Mohammad NF, Aurangzaib, Sharidan S (2012) Natural convection boundary layer flow of a viscoelastic fluid on solid sphere with Newtonian heating. World Academy of Science, Engineering and Technology (64): 628–633.

[27] Chaudhary RC, Jain P (2006) Unsteady free convection boundary layer flow past an impulsively started vertical surface with Newtonian heating. Romanian Journal of Physics (51): 911–925.

[28] Mebine P, Adigio EM (2009) Unsteady free convection flow with thermal radiation past a vertical porous plate with Newtonian heating. Turkish Journal of Physics (33): 109–119.

[29] Narahari M, Ishak A (2011) Radiation effects on free convection flow near a moving vertical plate with Newtonian heating. Journal of Applied Sciences (11): 1096–1104.

[30] Abid H, Khan I, Sharidan S (2013) An exact analysis of heat and mass transfer past a vertical plate with Newtonian heating. Journal of Applied Mathematics (2013): 1–9.

[31] Abid H, Ismail Z, Khan I, Hussein AG, Sharidan S (2014) Unsteady boundary layer MHD free convection flow in a porous medium with constant mass diffusion and Newtonian heating. The European Physical Journal Plus (129): 1–16.

[32] Abid H, Anwar MI, Farhad A, Khan I, Sharidan S (2014) Natural convection flow past an oscillating plate with Newtonian heating. Heat Transfer Research (45): 119–137.

[33] Jain A (2014) Chemically reactive boundary layer flow past an accelerated plate with radiation and Newtonian heating. International Journal of Engineering Research and General Science (20): 6–22.

